# Two Years versus One Year of Tianjiu Therapy in Sanfu Days for Chronic Asthma: A Clinical Efficacy Observation Trial

**DOI:** 10.1155/2014/807598

**Published:** 2014-09-11

**Authors:** Li Bing Zhu, Wei Zhang, Vivian Wong, Ziea Eric, Kwai Ching Lo, Wai Chung Chan, To Yau, Lei Li

**Affiliations:** ^1^School of Chinese Medicine, The University of Hong Kong, 10 Sassoon Road, Pokfulam, Hong Kong; ^2^Chinese Medicine Department, Hospital Authority, Hong Kong

## Abstract

*Background.* Tianjiu therapy has established efficacy against chronic asthma with related symptoms or the medication need during asthma attack. This study aimed to explore the optimal duration of Tianjiu therapy for asthma.* Methods.* This study was a self-comparison-to-the-baseline study, which comparing treatment with Tianjiu therapy for 1 year and 2 years in the same 102 chronic asthma patients. Totally 6 sessions of Tianjiu treatment were provided, 3 sessions in a year as a course of treatment and totally two years treatment. The primary endpoint was the number of asthma related symptoms which frequently appeared in asthma patients and the frequency of bronchodilator used during asthma attack.* Results.* The frequency of bronchodilator used during asthma attack significantly improved (*χ* ^2^ = 46.276, *P* = 0.000). But the number of asthma related symptoms which frequently appeared in asthma patients added by 1.38 points (95% CI, 0.25 to 2.51), 2.93 ± 0.41 in 1-year group and 4.31 ± 0.41 in the 2-years group (*P* < 0.05).* Conclusions.* The effect of 2 years Tianjiu therapy was not as effective as 1 year such treatment for asthma, but the second year Tianjiu therapy was still needed because it has a role to consolidate the curative effect of Tianjiu therapy for asthma.

## 1. Introduction

Asthma is one of the most common chronic diseases in the world. Currently, *β*-agonist as the first-line drug in treating acute asthma attack and glucocorticoids is still used widely to treat chronic asthma [1]. From the perspective of immunology, asthma is a systemic allergic disease with immune disturbance. Allergic airway inflammation is only a local show, and inhaled glucocorticoids also only pay attention to the local anti-inflammatory therapy while overlooking the systemic immune dysfunction in asthma patients. Hence, current therapy for asthma is still imperfect; it needs us to keep improving the therapy for asthma, and immunotherapy is likely to be one of the important ways to improve asthma therapy [2]. Traditional Chinese medicine (TCM) has a long history of treating asthma and it claimed that the mechanism of asthma mainly lies in two aspects: one is “deficiency in origin” and another is “enrichment in symptom.” The saying of “deficiency in origin” is similar to “systemic immune dysfunction,” and “enrichment in symptom” is similar to “allergic airway inflammation.” TCM pays more attention to the “preventive treatment of disease,” which means conduct prevention treatment for asthma patients when there is no asthma attack. Tianjiu Therapy in Sanfu Days is a classic prevention treatment for asthma. Sanfu Days means the three hottest days in a year which are calculated by ancient calendar. Both Positive-qi in human body and nature are in a most exuberant status in Sanfu Days, so Sanfu Days is a good time for cold-insufficiency patients to tonic Positive-qi. As a result, patients can have a strong body-resistance to against exogenous pathogen because they have already accumulative enough Positive-qi inside. Just as Chinese medicine said: “when there is sufficient Positive-qi inside, the Pathogenic-qi has no way to invade the health body.” Tianjiu Therapy means applying herbs patches on special acupoints in order to stimulate skin to form blisters, hyperemia, and even suppuration. As a result, Sanfu Tianjiu Therapy can attain the goal of strengthening Yang-qi, removing cold pathogen, and enhancing body resistance through the combination effect of drug infiltration absorption, acupoints stimulation, and time effect. Sanfu Tianjiu Therapy for asthma aims to improve the body immunity which in turn can get a purpose of preventing and reducing respiratory viral or bacterial infection, reducing airway inflammation injury, and reducing airway hyperresponsiveness which in turn reduces the times of asthma attacks [3–12]. Tianjiu Therapy substantially improves asthma control level and the quality of life in patients with asthma and is used widely in Mainland China as an adjuvant setting [3–12]. We have already conducted an initial trial which compared 1 year of Tianjiu treatment with a placebo control group. In that study, it was found that Tianjiu Therapy can really reduce the need for medications to control asthma, improved the quality of participants' life, and significantly reduced the level of asthma. Previous study has preliminary confirmed the effect of Tianjiu Therapy in Sanfu Days for asthma, while the optimal treatment duration is still unknown. Hence this study was conducted to compare the different effect of 2-year Tianjiu Therapy with 1-year such treatment in asthma patients.

## 2. Methods

### 2.1. Enrollment Criteria

This study was a self-control and clinical efficacy observation trial in 102 Hong Kong citizens (above 13 years of age) with chronic asthma. Participants need to provide physician's diagnosis of asthma or documentation of asthma-related symptoms preceding study visit. Besides, all participants should provide evidence of asthma attack as indicated by hospitalization or unscheduled urgent care twelve months before study entry. In addition, all patients were required to meet the following inclusion criteria: cough (worse at night), difficulty in breathing, chest tightness, awakening the patient, and symptoms occurring or worsening with exercise or at night; episodic symptoms of airflow obstruction, viral infections, strong emotions, and changes in weather. Patients would be assessed ineligibility according to one or more of the following exclusion criteria: severe cardiac and pulmonary diseases; fever, pharyngitis, acute asthma attack, and pregnancy. Diabetes mellitus, tuberculosis, and hypersensitive skin condition were also forming the excluded reasons. Additionally, allergy to topical medication, severe heart diseases, keloid, bleeding disorders or participants with pacemaker will also be excluded.

This study was approved by the Institutional Review Board of the University of Hong Kong/Hospital Authority Hong Kong West Cluster (HKU/HA HKW IRB) with coding UW 13-316. All participants were informed about this study and written informed consent was obtained from every patient or patient's legal guardian or the patient's parent.

### 2.2. Study Design

In this study, participants from 2010 to 2013 who totally receive two courses of Tianjiu Therapy (one course means completing three-time Tianjiu Therapy in a year, so two-course treatment needs at least two years and six-time Tianjiu Therapy) will constitute study subjects for final analysis. For example, patients who have already received 1-course Tianjiu Therapy in 2010 need to receive another course treatment in 2011 or 2012 or 2013. Patients from placebo group in 2010 still need to receive other 2-course treatment in the following three years. As a result, 102 patients who totally received two-course treatment were included for the final analysis. These 102 patients must complete two-course preassessment and postassessment which means that they have completed data in four time points. Among these 102 patients, there were 68 continuous participants (attended in 2010 and 2011, 2011 and 2012, or 2012 and 2013) plus 34 discontinuous participants (attended in 2010 and 2012, 2010 and 2013, or 2011 and 2013). This study conducted a subgroup analysis for continuous group and discontinuous group, but nothing different was found in most of the outcome measurements. Hence, this study combined continuous participants with discontinuous participants as study subjects.

Each year Asthma Control Test (ACT), self-made questionnaire, and pulmonary function will be assessed for participants before treatment and after treatment. Also three-time Tianjiu Therapy with 2-hour duration in a year was conducted for all participants from 2010 to 2013 and every time Tianjiu Therapy was conducted in Sanfu Days. In other words, eligibility patients participated in a certain year study then that year's three treatments and pre and post assessment were conducted for them. The three treatment times from 2010 to 2013 were July 19, July 29, and August 8, 2010; July 14, July 24, and August 13, 2011; July 18, July 28, and August 7, 2012; July 13, July 23, and August 12, 2013.

**Figure 1:**
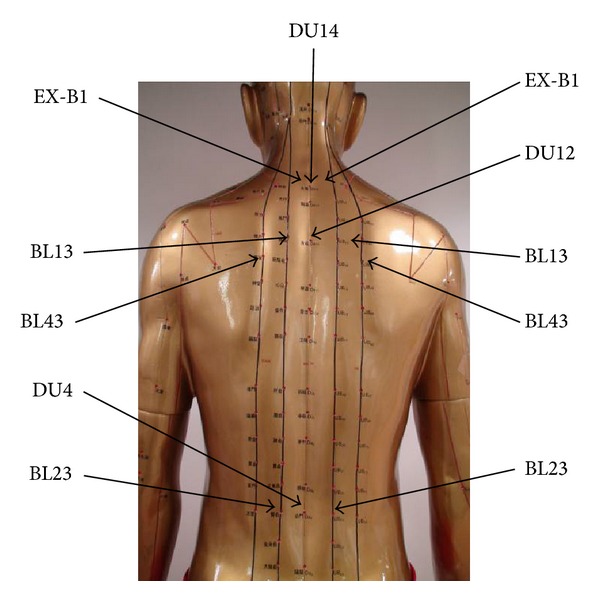
Acupoints.

Eleven acupoints will be applied by Tianjiu patches: BL13 (both sides), BL23 (both sides), BL43 (both sides), EX-B1 (both sides), Du14, Du12, and Du4 (Figure 1).

**Figure 2:**
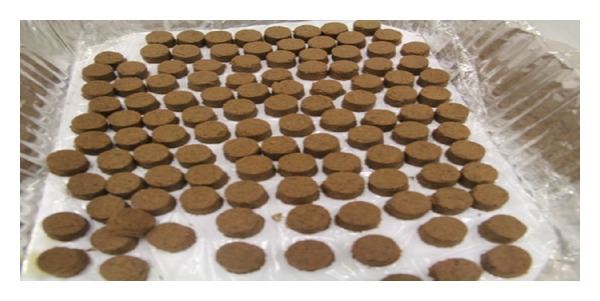
Tianjiu patch.

One patch was applied on one acupoint and every patch weighted 2 grams (Figure 2).

**Figure 3:**
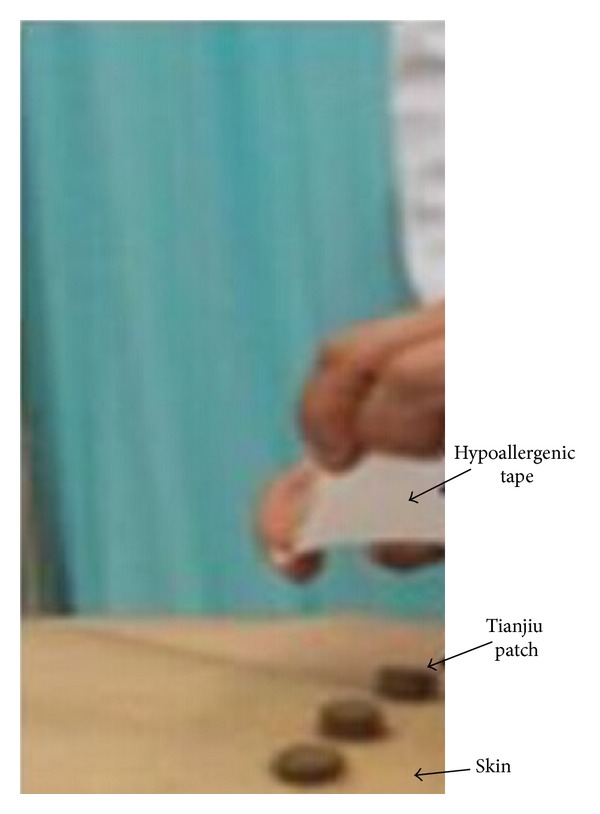
Tianjiu Therapy schematic diagram.

The formula of Tianjiu patch was a combination of the formula frequency used in clinical trials and the record in “Zhang Shi Yi Tong.” The final formula used consisted of* Sinapis alba, Radix Corydalis Yanhusuo, processed Euphorbia kansui, Asari Herba cum Radice, Ephedrae Herba, processed Radix Aconiti Praeparata, Cinnamomum cassia,* and* Eugenia caryophyllata*. The ratio of selected herbal was 2 : 1 : 1 : 1 : 1 : 1 : 1 : 1 and all this herbal powder will be mixed together with ginger juice. Finally, approximately 4 cm × 4 cm hypoallergenic tape (3M Micropore Tape 1535-3) will be used to stick Tianjiu patches on skin after the patch was applying on acupoints (Figure 3).

### 2.3. Outcome Measures

The primary outcome was the number of asthma-related symptoms which frequently appeared in asthma patients and the frequency of bronchodilator used during asthma attack. Other outcomes included the number of days with asthma-related symptoms in 1 month before visit; spirometric measurements; asthma-related health care use and the score on the ACT. Scores on the ACT range from 5 to 25, with scores of 20 or more indicating disease control and 3 points are the minimally important difference for the ACT score [13].

### 2.4. Statistical Analysis

Continuous variables in this study from 1st year baseline to 2nd year posttreatment were examined using a linear mixed-effects model with time as fixed effects. There were totally four time points in this linear mixed-effects model: 1st year baseline as the 1st time point, 1st year posttreatment as the 2nd time point, 2nd year baseline as the 3rd time point, and 2nd year posttreatment as the 4th time point. The continuous variables included the score of ACT, the number of asthma-related subhealthy symptoms, the number of days with asthma-related symptoms, and lung function: FEV and FEV_1_/FVC (%). All analysis was conducted for the same 102 people. Generalized estimated equation (GEE) analysis was performed for the frequency of bronchodilator used during asthma attack and asthma-related health care used during asthma attack in order to repeat measures in different time points. Descriptive analysis was conducted for the percentage of patients with twenty-three asthma-related symptoms which frequently appeared in asthma patients. Statistical analyses were performed with the Statistical Package for the Social Sciences (SPSS) software program (version 19) for windows XP.

## 3. Results

From June 2010 through July 2010, 323 patients were recruited in 2010 and finally 242 patients completed treatment. 274 patients were recruited as study subjects in 2011, of which 84 participants were from 2010 Tianjiu Therapy group, 82 were from 2010 placebo group, and 108 were new recruited subjects. During the treatment period and postassessment in 2011, 104 patients did not complete all parts. As a result, 170 participants completed all treatments and postassessment in 2011. Totally 132 patients completed all treatments and pre- and postassessments in 2012, and 150 patients lastly completed preassessments, postassessments, and three-time Tianjiu Therapy in 2013. Patients would be ineligible for final statistical analysis due to the incomplete data. The cause of incomplete data included incomplete treatments, during treatment period participants losing contact, illnesses, pregnancy, asthma exacerbation, inability to participate timely, refusing to participate, or incomplete questionnaires, ACT, or lung function test. The patients can be included for final analysis only when they have four time points' data (1st year pre- and postassessments, 2nd year pre- and postassessments). Overall, from 2010 to 2013, patients who received two-course treatment were 102.

For the baseline characteristics of these 102 participants, the average age was 50.4 years (interquartile range, 13 to 78); 53% were female (Table 1). The average number of days on which patients had asthma-related symptoms was 9.35. Patients had a mean ACT score of 19 or less which indicated a lack of disease control [14]. In the previous year, 22.5% patients received emergency treatments at least once which were associated with an asthma-related event, 18.6% had been hospitalized, 58.8% went to clinic, 70.6% treated themselves following the prescription, 10.8% buy medication for themselves, and 9.8% did not need medical treatment. The mean (±SD) FEV_1_ was 1.86 ± 0.98, and the mean ration of FEV_1_ to the forced vital capacity (FVC) was 85.24 ± 27.76. For the frequency of bronchodilator used during asthma attack, 24.5% used bronchodilator more than twice per day, 37.3% once to twice per day, 4.9% 2-3 times per week, and 15.7% less than once per week, and 17.6% never used bronchodilator during asthma attack. The number of symptoms which frequently appeared in asthma patients was 8.20 ± 4.40.

### 3.1. Response to Intervention

**Figure 4:**
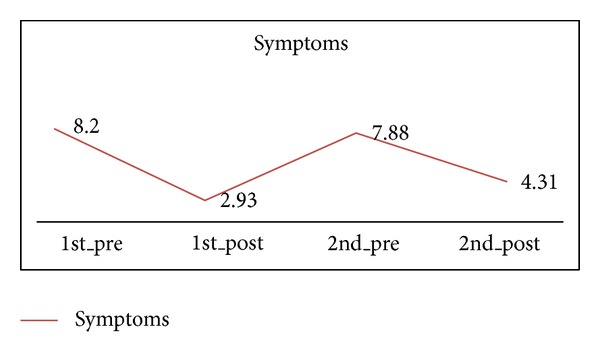
The number of symptoms which frequently appeared in asthma patients in 1 course and 2 courses.

The number of symptoms which frequently appeared in asthma patients was 2.93 ± 0.41 in end of 1-course treatment and 4.31 ± 0.41 in end of 2-course treatment; one course has 5.27-point reduction and 2 courses have 3.88-point reduction, respectively, comparing with 8.20 ± 0.41 in baseline (*P* < 0.05) (Figure 4 and Table 2). But 1-course treatment reduced more points than 2-course treatment (*P* < 0.05).

**Figure 5:**
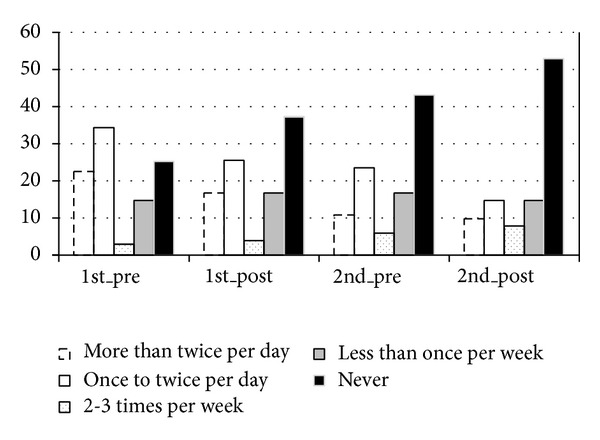
The frequency of bronchodilator used during asthma attack.

The frequency of bronchodilator used during asthma attack in 2 courses was better than 1 course: 16.7% more than twice per day in 1 course and 9.8% in 2 courses, 25.5% once to twice per day in 1 course and 14.7% in 2 courses, 3.9% 2 to 3 times per week in 1 course and 7.8% in 2 courses, and 16.7% less than once per week in 1 course and 14.7% in 2 courses, and 37.3% never used it in 1 course and 52.9% in 2 courses, indicating that the frequency of bronchodilator used during asthma attack was decreasing (Table 3 and Figure 5, *P* < 0.05).

**Figure 6:**
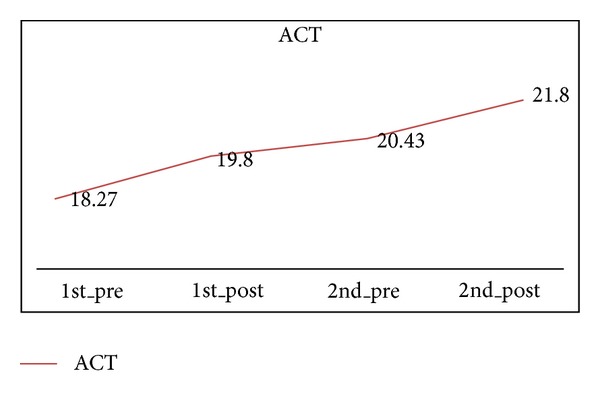
The score of ACT in 1 course and 2 courses.

The score of ACT improved by 1.53 points (95% CI, −0.31 to 3.37) after one-course treatment (19.80) and 3.53 points (95% CI, 1.69 to 5.37) after two courses of treatment (21.80) (Figure 6 and Table 4). In other words, the score of ACT after two-course treatment was better than one-course treatment and the score of ACT showed an increasing trend (*P* < 0.05).

**Figure 7:**
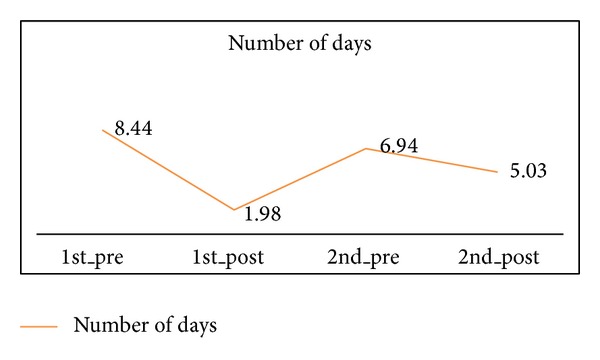
The number of days with asthma-related symptoms in 1 year and 2 years.

Different from the improvement like the score of ACT and the frequency of bronchodilator used during asthma attack, as compared with 1-year treatment, 2-year treatment with Tianjiu increased the mean number of days on which patients had asthma-related symptoms from 1.98 to 5.03 (*P* < 0.05) (Table 2 and Figure 7); even both 2-year treatment and 1-year treatment compared with baseline have a significant improvement (*P* < 0.05).

No changes occurred in pulmonary function for both 1-year treatment and 2-year treatment comparing with no treatment status. With respect to asthma-related health care use during asthma attack, the percentage of participants admitted to Accident and Emergency Departments (A and E) was 1% in year 1 and 6% in 2 years; comparing with baseline both have a significant improvement (*P* < 0.05). Similarly, the proportion of patients who were hospitalized due to asthma was 0% in 1 year and 2% in 2 years, with both being better than no treatment (*P* < 0.05). The percentage of participants who visited outpatient clinic was 9% in 1 year and 49% in 2 years; both of them have a significant improvement in comparison with no treatment; however, the effect of 1 year was much better than 2 years (*P* < 0.05). The ratio of subjects who took the previous prescription by doctors during asthma attack was 60% in 1 year and 69% in 2 years, and no difference occurred in both groups comparing with no treatment (*P* > 0.05); the scale of patients who only need to take medication by themselves was 1% in 1 year and 8% in 2 years; 1-year treatment was better than no treatment while 2-year treatment was not different from baseline, indicating that 1-year treatment was better than 2-year treatment (*P* < 0.05); last but not least, the percentage of patients who do not need to process during asthma attack was 2% in 1 year and 10% in 2 years; 2-year treatment has nothing different from baseline, while 1-year treatment was worse than no treatment and 2 years performed better than 1 year (*P* < 0.05) (Table 2).

**Figure 8:**
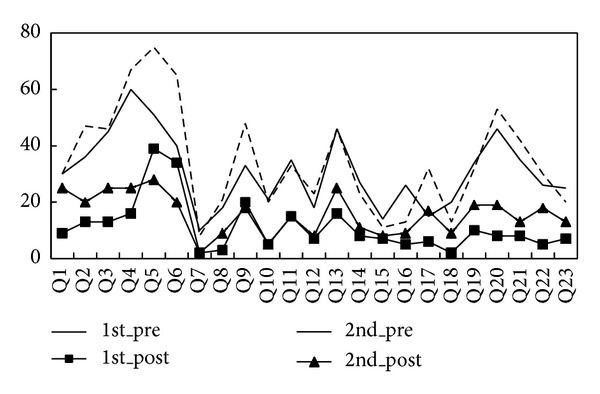
The proportion of participants with twenty-three symptoms which frequently appeared in asthma patients in 1 course and 2 courses.

Figure 8 showed the percentage of participants with twenty-three symptoms which frequently appeared in asthma patients. Among these twenty-three symptoms, the top three frequently occurring symptoms were as follows: easy onset during quarter turn (67% in baseline, 16% after 1-course treatment, and 25% after 2-course treatment), rapid or difficult breathing (75% in baseline, 39% after 1-course treatment, and 28% after 2-course treatment), and waking up with asthma symptoms (65% in baseline, 34% after 1-course treatment, and 20% after 2-course treatment), indicating that the majority of participants owned the lung-qi-deficiency syndromes. Nearly all patients have a significant reduction in the twenty-three symptoms after both 1-course Tianjiu Therapy and 2-course such treatment. What is more, it showed that the status of patients in the 2nd course baseline was better than 1st course baseline which indicated that Tianjiu Therapy has a cumulative effect after one course of treatment. Additionally, from Figure 8, it can be found that the reduction of 1 course was much more than 2 courses. It seems that the effect of 2 courses was not as sensitive as 1 course, regardless of the fact that 2-course treatment still has a significant improvement compared with 1st course baseline.

### 3.2. Adverse Event

The observation procedures followed after Tianjiu Therapy were conducted in both 1 course and 2 courses. During the treatment, most patients would show cutaneous reaction such as skin warm feeling, itching, pain, and blisters due to the stimulation of herbal patch. All these appearances were a normal skin reaction after Tianjiu Therapy and also a key condition of Tianjiu Therapy taking effect in the treatment of asthma. Most of them would disappear after several hours or several days. In 1st course Tianjiu Therapy, there were five serious adverse events reported at the end of three-time treatment: three patients showed asthma exacerbation, one patient showed the rash due to being allergic to Tianjiu patch, and one subject tended to vomit blood and automatically stopped after the 2nd Tianjiu Therapy, which had confirmed that it has nothing to do with Tianjiu Therapy. Besides, in 2nd course Tianjiu Therapy, the majority of patients also showed skin reaction after Tianjiu Therapy as that in 1st course. There were also five serious adverse events in the 2nd course treatment: three were skin sensibility which cannot disappear after stopping treatment even several days later. And finally, his skin returned to normal through dermatologist's appropriate process. one person was caused by the smell of paint because his house was mopping during the treatment period; another was induced by flu. Overall, there was nothing different between 1st course Tianjiu Therapy and 2nd course Tianjiu Therapy in adverse events.

## 4. Discussion

In the previous study (HKCTR-1128), Tianjiu Therapy has shown substantial improvements in the number of days with asthma-related symptoms, the number of symptoms which frequently appeared in asthma patients, and the medication need after treatment. Although there was nothing different between placebo group and Tianjiu Therapy group after the 3rd treatment immediately, the effect of Tianjiu Therapy tended to be superior to placebo group in four-time followup. This study was conducted to explore how long the curative effect of Tianjiu Therapy lasts and the optimum duration of treatment for chronic asthma based on previous finding. This study tested the hypothesis that continuation of Tianjiu Therapy beyond 1 course would be more effective than 1 course of such treatment. As a result, this study found that two courses of Tianjiu Therapy showed a significant improvement in the score of ACT as compared with 1-course treatment and the score of ACT showed an increasing trend. In addition, the frequency of bronchodilator used during asthma attack in 2 courses was also superior to the effect of 1 course. Patients' asthma control situation in 2nd pretreatment was better than that in the 1st pretreatment. Which means patients' immunity improved and kept well after one-course Tianjiu Therapy? While, it was arbitrary to make conclusion that the effect of 2-course Tianjiu Therapy was superior to 1 course by only judging from the change of the score of ACT and the bronchodilator used during asthma attack between 1 course and 2 courses. Nevertheless, different from the findings above, the result of the mean number of days on which patients had asthma-related symptoms shows no such additional benefit; the effect of 2-course treatment was close to 1 course such treatment. Likewise, results about the number of symptoms which frequently appeared in asthma patients, asthma-related health care use during asthma attack, and the percentage of participants with symptoms which frequently appeared in asthma patients were not mostly different between the 1-course treatment and 2-course such treatment. Last but not least, no changes occurred in pulmonary function for both 1-course treatment and 2-course treatment comparing with 1st course baseline.

Although the result was inconsistent in all outcome measurements, it was found that the effect of 2-course Tianjiu Therapy was less sensitive than 1 course of such treatment for chronic asthma, but both 1-course and 2-course Tianjiu Therapy can get a significant improvement comparing with the situation in 1st pretreatment. And the baseline status of the same people in the 2nd course remained well even one year later after the first course of treatment, indicating that the effect of Tianjiu Therapy can last for a quite long time. For safety issue, the number of adverse events in 2nd course treatment was nearly the same as the number in 1st course treatment. There is no doubt that all these findings will be beneficial for health policy makers and asthma patients.

There is a limitation of this study because this study did not set up followups and only takes the second course's baseline as the followup of first course treatment. However, Sanfu Days in every year were around July and August which was a stable period for asthma patients, so it was easy to produce bias without followup in asthma high incidence and research staff cannot track the asthma control situation of participants. Hence, it is urgent and necessary to add followups.  Table 5 showed the temperature record of 2010 to 2013. The average temperature in Sanfu Days was 28.7°C from 2010 to 2013; the average humidity was 81% from 2010 to 2013; the average duration of sun exposure was 6.6 hours from 2010 to 2013.

## 5. Conclusion

Both 2-course Tianjiu Therapy and one-course Tianjiu Therapy significantly reduced the number of symptoms which frequently appeared in asthma patients and medication need in participants with chronic asthma as compared with baseline. The asthma control situation in 2nd course pretreatment was better than 1st course pretreatment. Although the second course Tianjiu Therapy was less sensitive than first course Tianjiu Therapy for chronic asthma, the second course treatment still plays a role in consolidating the curative effect of Tianjiu Therapy in the treatment of asthma.

## Figures and Tables

**Table 1:** Baseline characteristics of study participants.

Characteristic	Tianjiu Therapy (*N* = 102)
Age—year	50.35 ± 17.17
Gender—number (%)	
Male	48 (47.1)
Female	54 (52.9)
Duration of asthma—year	20.22 ± 14.14
Asthma-related symptoms—number of^a^	9.35 ± 11.90
Lung function	
FEV_1_	1.86 ± 0.98
FEV_1_:FVC × 100	85.24 ± 27.76
Asthma-related health care—number (%)	
≥1 Admitted to A and E^b^	23 (22.5)
≥1 Hospitalization	19 (18.6)
≥1 Outpatient visit	60 (58.8)
≥1 Prescription	72 (70.6)
≥1 Buy medication	11 (10.8)
≥1 Not to be processed	10 (9.8)
The frequency of bronchodilator used during asthma attack—number (%)	
More than twice per day	25 (24.5)
Once to twice per day	38 (37.3)
2-3 times per week	5 (4.9)
Less than once per week	16 (15.7)
Never	18 (17.6)
Symptoms which frequently appeared in asthma patients—number of symptoms^c^	8.20 ± 4.40

^a^Plus-minus values are means ± SD, unless noted otherwise. PIF denotes peak inspiratory flow; PEF: peak expiratory flow; FEV_1_: forced expiratory volume in one second; FVC: forced vital capacity.

^b^The total number of symptoms which frequently appeared in asthma patients is 23 and its scale ranges from 0 to 23.

^c^Accident and Emergency Departments (A and E).

**Table 2:** Variable on asthma patients in 1 course and 2 courses.

Variable	1st baseline	1st posttreatment	2nd baseline	2nd posttreatment	1st posttreatment versus 1st baseline (95% CI)/*P* value	2nd posttreatment versus 1st baseline (95% CI)/*P* value	2nd posttreatment versus 1st posttreatment (95% CI)/*P* value
Asthma-related symptoms—number of days^a^	8.44 ± 0.99	1.98 ± 0.99	6.94 ± 0.99	5.03 ± 0.99	−6.46 (−9.21 to −3.72)^###^	−3.41 (−6.16 to −0.67)^#^	3.05 (0.30 to 5.80)^#^
Lung function							
FEV	1.86 ± 0.09	2.01 ± 0.09	1.80 ± 0.09	1.76 ± 0.09	0.15 (−0.11 to 0.40)	−0.10 (−0.36 to 0.15)	−0.25 (−0.51 to 0.003)
FEV_1_:FVC × 100	85.24 ± 1.87	84.72 ± 1.87	81.89 ± 1.87	84.56 ± 1.50	−0.52 (−5.70 to 4.67)	−0.68 (−5.86 to 4.51)	−0.16 (−5.35 to 5.03)
Symptoms which frequently appeared in asthma patients—number of symptoms^b^	8.20 ± 0.41	2.93 ± 0.41	7.88 ± 0.41	4.31 ± 0.41	−5.27 (−6.40 to −4.13)^###^	−3.88 (−0.50 to −2.75)^###^	1.38 (0.25 to 2.51)^#^
Asthma-related health care use—number (%)							
≥1 Admitted to A and E^c^	23 (23)	1 (1)	16 (16)	6 (6)	0.000^###^	0.000^###^	0.054
≥1 Hospitalization	19 (19)	0 (0)	11 (11)	2 (2)	0.000^###^	0.000^###^	0.153
≥1 outpatient visit	60 (59)	9 (9)	52 (51)	50 (49)	0.000^###^	0.000^###^	0.000^###^
≥1 Prescription	72 (71)	61 (60)	79 (77)	70 (69)	0.066	0.758	0.102
≥1 Buy medication	11 (11)	1 (1)	13 (13)	8 (8)	0.001^##^	0.437	0.006^##^
≥1 Not to be processed	10 (10)	2 (2)	15 (15)	10 (10)	0.018^#^	1.000	0.018^#^

^#^ *P* < 0.05; ^##^ *P* < 0.01; ^###^ *P* < 0.001.

^a^Plus-minus values are means ± SE, unless noted otherwise. PIF denotes peak inspiratory flow; PEF: peak expiratory flow; FEV_1_: forced expiratory volume in one second; FVC: forced vital capacity.

^b^The total number of symptoms which frequently appeared in asthma patients is 23 and its scale ranges from 0 to 23.

^c^Accident and Emergency Departments (A and E).

**Table 3:** The frequency of bronchodilator used during asthma attack—*n* (%).

Variable	Time			
	1st_pre	1st_post	2nd_pre	2nd_post
More than twice per day	23 (22.5)	17 (16.7)	11 (10.8)	10 (9.8)
Once to twice per day	35 (34.3)	26 (25.5)	24 (23.5)	15 (14.7)
2-3 times per week	3 (2.9)	4 (3.9)	6 (5.9)	8 (7.8)
Less than once per week	15 (14.7)	17 (16.7)	17 (16.7)	15 (14.7)
Never	26 (25.2)	38 (37.3)	44 (43.1)	54 (52.9)

Time main effect: Wald *χ* ^2^ = 46.276, *P* = 0.000.

**Table 4:** ACT score before and after treatment in 1 course and 2 courses.

1st baseline	18.27 ± 0.66
1st posttreatment	19.80 ± 0.66
2nd baseline	20.43 ± 0.66
2nd posttreatment	21.80 ± 0.66
1st posttreatment versus 1st baseline (95% CI)/*P* value	1.53 (−0.31 to 3.37)
2nd posttreatment versus 1st baseline (95% CI)/*P* value	3.53 (1.69 to 5.37)^###^
2nd posttreatment versus 1st posttreatment (95% CI)/*P* value	2.00 (0.16 to 3.84)^#^

Notes: ^#^ *P* < 0.05; ^###^ *P* < 0.001.

**Table 5:** Temperature record in Sanfu Days from 2010 to 2013.

Item	Year											
	2010			2011			2012			2013		
	1st hottest day (July 19)	2nd hottest day (July 29)	3rd hottest day (Aug. 8)	1st hottest day (July 14)	2nd hottest day (July 24)	3rd hottest day (Aug. 13)	1st hottest day (July 18)	2nd hottest day (July 28)	3rd hottest day (Aug. 7)	1st hottest day (July 13)	2nd hottest day (July 23)	3rd hottest day (Aug. 12)
Maximum temperature (°C)	31.3	31.2	31.4	31.6	31.3	30.9	31.4	31.2	31.3	31.6	31.3	30.9
Temperature (°C)	28.8	28.6	28.8	28.9	28.8	28.4	28.8	28.6	28.8	28.9	28.8	28.5
Minimum temperature (°C)	26.8	26.6	26.8	26.8	26.9	26.4	26.8	26.7	26.8	26.8	26.8	26.5
Relative humidity (%)	81	82	82	80	80	83	81	81	81	80	81	83
Sun exposure (hour)	6.7	6.1	6.5	7.5	7.1	5.8	6.7	6.1	6.6	7.5	7.3	5.8
